# Editorial: Inequality in healthcare utilization and household spending in developing countries

**DOI:** 10.3389/fpubh.2022.970819

**Published:** 2022-08-09

**Authors:** Mihajlo Jakovljevic, Asankha Pallegedara, Thanabalasingam Vinayagathasan, Ajantha Sisira Kumara

**Affiliations:** ^1^Institute of Advanced Manufacturing Technologies, Peter the Great St. Petersburg Polytechnic University, St Petersburg, Russia; ^2^Institute of Comparative Economic Studies, Hosei University, Tokyo, Japan; ^3^Department of Global Health Economics and Policy, University of Kragujevac, Kragujevac, Serbia; ^4^Department of Industrial Management, Faculty of Applied Sciences, Wayamba University of Sri Lanka, Kuliyapitiya, Sri Lanka; ^5^Department of Economics and Statistics, Faculty of Arts, University of Peradeniya, Peradeniya, Sri Lanka; ^6^Department of Public Administration, Faculty of Management Studies and Commerce, University of Sri Jayewardenepura, Nugegoda, Sri Lanka

**Keywords:** equality, healthcare utilization, out-of-pocket health expenses, developing countries, health policy

Ensuring equitable utilization of healthcare services for those in need is vital to reduce financial risks associated with rising healthcare expenditure in both developed and developing countries. When presenting Sustainable Development Goals, the United Nations clearly link equitable utilization of healthcare services to the health status and overall wellbeing of people. However, socio-economic inequalities in healthcare utilization and out-of-pocket health expenses have increased in countries, creating an intense political pressures on respective governments to have higher budgetary allocations for healthcare. The situation in low-and middle-income countries is even worse as their uneven distribution of healthcare infrastructure has aggravated the situation. This has resulted in degenerated-health outcomes, increased-morbidity and–mortality among deprived-groups in those countries. On this backdrop, examining the issues pertaining to inequality in healthcare utilization and out-of-pocket healthcare expenses is vital from public policy viewpoints. The studies may discuss inequality-related issues faced by countries depending on rich and updated-datasets and novel analytical tools. Accordingly, this special issue publishes six studies of that nature, contributing to the extant literature on the subject.

Tian et al. examined the degree of healthcare utilization of migrants in China and how it is influenced by health education. The study argues that health education plays a vital role in enhancing the degree of healthcare utilization among migrants in China, and thereby, health education generates promotional effects with regard to healthcare utilization. The study further finds that counseling is more effective than other methods of health education in this regard. Moreover, the study concludes that the promotional effects of health education on healthcare utilization is favorably moderated by the proximity to medical services: closer to the services, greater the promotional effects. Accordingly, one could expect that health education could play a significant role in mitigating inequality in healthcare utilization by migrants and non-migrants in China.

You et al. provide empirical evidence on inequality in consumption-related health education in China during COVID-19. The paper argues that health education could play a significant role in preventing a pandemic like COVID-19, and nonetheless, depending on socio-economic characteristics of individuals, there persists a considerable degree of inequality among different subgroups. The empirical evidence provided in terms of Concentration Index, Horizontal Index, and decomposition analysis show that health education is highly concentrated among the individuals with higher-level of general education, higher income, and more positive consumption preferences. Moreover, the study uncovers that inequality in health education among individuals is also caused by health status, type of residence, and medical insurance ownership. The results imply that the government needs to pay attention on vulnerable people including, the ones with lower-level of education and income and rural living when planning for health educational programs at the recovery stage of COVID-19.

The similar nature of analysis is provided by Zhang et al. with regard to healthcare utilization by the individuals in economically under-developed regions in Northeast China. The severe inequality in healthcare utilization among people in the aforesaid region is primarily associated with household income, health status, place of living, and health insurance ownership. The results imply policy changes to protect poor people from under-utilization of healthcare services. Moreover, improved-social security systems along with basic health and critical illness insurance would mitigate inequality in healthcare utilization of individuals living in economically backward Chinese regions.

Providing more specific empirical evidence on inequality in healthcare utilization among Chinese cancer patients, Wang et al. show that rural cancer patients report less-level of healthcare utilization including, screening and treatments compared to their urban counterparts. Thus, the study recommends upgrading rural-sector healthcare infrastructure required for cancer patients.

Using a cross-sectional dataset from China, Nie et al. develop a model to predict the level of utilization of Family Doctor Contract Services (FDCS). The study finds that individuals' age, gender, household income, educational attainment, insurance status, health condition, smoking and drinking habits, physical activity status, and some supply-side factors can be used to relevant predictions. Overall, the model exhibits moderate performance.

Xie et al. analyze the economic burden of neurodevelopmental diseases on Chinese patients with genetic diagnosis. The economic burden is computed by taking direct and indirect medical and non-medical cost associated with relevant healthcare utilization into account. As the insurance coverage is low among these patents, they need to bear a huge economic burden. The study proposes that early diagnosis is crucial in reducing this burden. Moreover, the paper emphasizes role of government in increasing insurance coverage to reduce the vulnerability of patents.

The Global South countries were represented in this Topic, present diverse legacy of Inequality in Healthcare Utilization and Household Spending. LMICs countries health systems experience difficult sustainability challenge due to long-term trends (1). Some of their bottleneck vulnerabilities are exposed only in circumstances where entire health sectors are pushed to the limits of their resilience (2). Asia—Pacific region, China, India, Sri Lanka and South-East Asian ASEAN market remain the strongest drivers of wide regional real economic growth (3).

Despite heavy emphasis on South Asian and ASEAN health systems in this Topic, other world regions might expose dynamic development as well with an impact to global Inequality in Healthcare Utilization and Household Spending (3). Convenient example is Eastern Europe. These countries might have in common with nations such as Cambodia, Laos and Vietnam legacy of real-socialism, concept of social justice and were mostly centrally planned economies during the Cold War Era (3). In addition, many of them were effectively grounded in Soviet Semashko tradition of health care establishment and provision of medical services (4). Many others in central Europe and former Yugoslavia also share a legacy of mixed Bismarck system. These practices faced with multiple waves of health care reforms during the past three decades have created numerous social tensions (5). In particular rise of inequalities in terms of equity and affordability of increasing private health care provision facilities in some areas such as dentistry, gynecology clinics of esthetic surgery (6). New rich elites of the Balkans and Eastern Europe, situated mostly in metropolitan areas are contrasted to poor rural countryside without effective access to secondary and tertiary care (7). This is the widespread phenomenon ranging from Poland and Hungary to Bulgaria, Serbia and Baltic states.

Heterogeneous Topic contributions could reveal a new knowledge frontier. Global South countries challenges from the perspective of academic health economics (8). The goals of this special edition were to explore underlying patterns of inequality in healthcare utilization and household spending across this vast region. Stakeholders involved in these challenges including pharmaceutical and medical device industry (9), governments, universities, physician chambers and patient associations took part in this debate. Editors believe that this article collection could provide hints for plausible further avenues of scientific endeavor in foreseeable future.

## Author contributions

MJ has drafted the manuscript while AP, TV, and AK have expanded it substantially for important intellectual content. All authors have equally contributed to this editorial fulfilling ICMJE criteria for full authorship. All authors contributed to the article and approved the submitted version.

## Conflict of interest

The authors declare that the research was conducted in the absence of any commercial or financial relationships that could be construed as a potential conflict of interest.

## Publisher's note

All claims expressed in this article are solely those of the authors and do not necessarily represent those of their affiliated organizations, or those of the publisher, the editors and the reviewers. Any product that may be evaluated in this article, or claim that may be made by its manufacturer, is not guaranteed or endorsed by the publisher.
